# Rapid urine screening for ethyl glucuronide from pregnant women as a tool for detecting prenatal alcohol exposure

**DOI:** 10.1186/s12884-023-05789-x

**Published:** 2023-06-22

**Authors:** Mirjami Jolma, Mikko Koivu-Jolma, Onni Niemelä, Ilona Autti-Rämö, Hanna Kahila

**Affiliations:** 1 Division of Child Neurology, Päijät-Häme Central Hospital, Keskussairaalankatu 7, 15850 Lahti, Finland; 2 Faculty of Medicine, The University of Helsinki, Haartmaninkatu 8, P.O. Box 63, 00014 University of Helsinki, Finland; 3 Faculty of Science, The University of Helsinki, Gustaf Hällströminkatu 2, P.O. Box 64, 00014 University of Helsinki, Finland; 4 Medical Research Unit, Seinäjoki Central Hospital and University of Tampere, Tampere, Finland; 5 Department of Obstetrics and Gynecology, University of Helsinki and Helsinki University Hospital, Haartmaninkatu 2, PO Box 22, 00014 Helsinki, Finland

**Keywords:** Ethyl glucuronide, Prenatal alcohol exposure, Urine, Screening

## Abstract

**Background:**

An increasing prevalence of alcohol consumption is a major public health problem, which has also led to an increasing number of children who have been prenatally exposed to the toxic effects of ethanol. However, obtaining reliable information on prenatal alcohol exposure through maternal self-reports has proved difficult.

**Aims:**

Our aim was to evaluate the potential for rapid screening test for measuring ethyl glucuronide (EtG), a specific alcohol metabolite, from urine samples of pregnant women.

**Methods:**

Five hundred five urine samples of pregnant women were collected anonymously from five prenatal units in two Finnish cities: a tertiary specialist antenatal clinic for pregnant women with problematic substance use (HAL), a regular hospital antenatal clinic (LCH = Lahti Central Hospital), a prenatal screening unit and two community maternity clinics (USR = user self-recruiting units). All samples were screened using rapid EtG test strips, and all positive, uncertain, and randomly selected negative samples were confirmed by quantitative analyses. The samples were also screened for cotinine and use of cannabis.

**Results:**

In this material an EtG cut-off of 300 ng/mL suggesting heavy alcohol drinking was exceeded by 7.4% (5/68) of the samples in the HAL clinic, 1.9% (4/202) in LCH, and 0.9% (2/225) in USR. A cut-off of 100 ng/mL was exceeded by 17.6% (12/68) of samples from HAL, 7.5% (16/212) from LCH, and 6.7% (15/225) from USR. Based on confirmatory quantitative analyses, there were no false negatives nor false positives in rapid EtG screening. However, 57 (11.3%) of test results were classified as uncertain. In these cases, confirmation by quantitative analyses resulted in 56.1% rate of positive values. 73% of the samples with EtG > 300 ng/mL showed positive cotinine results suggesting smoking co-occurring with alcohol intake.

**Conclusions:**

Rapid EtG tests may be an easy and inexpensive method, which may improve the possibilities for screening alcohol use among pregnant women during routine prenatal visits. Quantitative EtG analyses are recommended to confirm screening positive and uncertain cases.

**Trial registration:**

NCT04571463 Date of Registration 11/05/2020.

## Background

Prenatal alcohol exposure (PAE) may lead to a wide spectrum of phenotypic alterations called Fetal Alcohol Spectrum Disorders (FASD) ranging from Alcohol-Related Neurodevelopmental Disorder (ARND) to fully developed fetal alcohol syndrome (FAS) [1, 2]. It is currently recommended to fully abstain from alcohol use during pregnancy according to both WHO [3] and Finnish national recommendations [4] However, we do not know how well women follow this recommendation since self-reports on alcohol consumption during pregnancy tend to be less reliable than self-reports on drinking habits in general [5].

Alcohol use among women is a major public health problem worldwide. In Finland, recent estimates have indicated that 84% of women aged 20–44 years drink alcohol and 3% report heavy weekly binge-drinking (the Finnish national definition for binge drinking is ≥ 6 alcohol units on one occasion, 1 alcohol unit in Finland equals 12 g of pure ethanol). Furthermore, 24% of women aged 20–34 years were considered to use alcohol regularly in amounts regarded as excessive (more than 5/12 points in AUDIT-C) [6].

According to a European study 14% of Finnish women anonymously reported alcohol consumption after awareness of pregnancy [7]. In one Finnish anonymous survey more than half of pregnant women reported some alcohol use during pregnancy and almost 5% reported binge drinking [8] according the to the Finnish definition (≥ 72 g of pure ethanol on one occasion). In a recent cohort study of 14 822 pregnant Finnish women based on self-reporting via electronic questionnaires 26% reported stopping alcohol use only after recognizing being pregnant and 4.6% self-reported continuing alcohol use during pregnancy [9]. A German study showed that when asked about alcohol use during pregnancy or later, women tend to underreport or deny it, even when the child’s meconium shows evidence of alcohol metabolites [10].

The absence of documented prenatal alcohol exposure makes diagnosis of FASD difficult if dysmorphic facial features are absent, even when the child shows typical signs of neurobehavioral problems [1]. Only a minority of children with FASD show signs of dysmorphic FAS [11, 12]. According to health records, diagnoses of FASD are rare in Finland, about 4.5/10 000 (personal communication Mika Gissler Finnish Institute for Health and Welfare). In countries where women’s alcohol consumption is similar to that in Finland, the prevalence of FASD is 2–7% depending also on the criteria used to define the phenotype [12-14]. Therefore, it is important to investigate prenatal alcohol exposure also using objective laboratory tests, and the need for rapid diagnostic methods for recognizing fetal alcohol exposure has been widely acknowledged [15, 16].

Ethyl glucuronide (EtG) is a specific metabolite of ethanol that is excreted to urine and can be found in urine samples up to several days after consumption of large amounts of alcohol [17] and about 24 h after smaller alcohol amounts [18].

When examining alcohol use disorders, a cut-off of 500 ng/mL has been suggested [19]. A lower cut-off during pregnancy may, however, be rational because fetal tissues lack capacity for clearance of ethanol and its toxic metabolites. There is no known safe amount of alcohol use during pregnancy and during pregnancy, a cut-off of 100 ng/mL has been previously used [20, 21].

Combined tobacco and alcohol exposure during pregnancy exacerbates the damage caused by alcohol [22]. Alcohol use and smoking have been shown to be highly concomitant behaviors during pregnancy in Canada [23] and Norway [24]. It is important to know if that applies also in Finland, where 10% of women smoke during pregnancy [25].

### Aims

The aims of this study were:

- 1. to evaluate the possible usefulness of rapid EtG testing as a tool for detecting ethanol exposure during pregnancy.
- 2. to assess the prevalence of positive EtG values in different prenatal clinics.
- 3. to examine whether smoking status, cannabis use, gestational age, or timing of sampling during the week (early or late) correlates with the number of positive EtG findings.

## Methods

Pregnant women were recruited between 1^st^ of October 2020 and 4^th^ of June 2021 from five clinics in two cities, Helsinki (Finnish capital with ca 550 000 inhabitants) and Lahti (9th largest city in Finland with ca 120 000 inhabitants). The clinics and their sample collection schedules were as follows:

- 1. HAL Clinic a tertiary specialist antenatal clinic for pregnant women with problematic substance use at Women’s Hospital in Helsinki. Collection time: from 1^st^ of October to 1^st^ of December 2020 and from 12^th^ of January to 7^th^ of May 2021. Recruitment by midwives.
- 2. Lahti central hospital specialist antenatal clinic (LCH). Collection time from 1^st^ of November to 11^th^ of December 2020. Recruitment by midwives.
- 3. Prenatal ultrasound screening unit at Women’s Hospital in Helsinki. Collection time from 1^st^ October to 30^th^ of November 2020. A user self-recruiting unit (USR).
- 4. Primary care maternity clinic in Helsinki. Collection time from 26^th^ March to 4^th^ of June 2021. A user self-recruiting unit (USR).
- 5. Primary care maternity clinic in Lahti. Collection time from 15^th^ of March to 28^th^ of May 2021. A user self-recruiting unit (USR).

To achieve anonymity, the personnel in antenatal clinics recruited participants for the study only by informing the visitors of the study and describing the participation procedure. Additional information, if required, was available through dedicated mobile phone and email address. Participation rate in the specialist antenatal clinics was calculated by dividing the number of samples received by the number of pregnant patients that were informed of the study by midwives.

In primary care maternity clinics both in Lahti and Helsinki, and prenatal ultrasound screening unit, women were anonymously self-recruited. An advertisement of the study was sent attached to a standard clinic invitation letter. Self-recruitment was based primarily on the specifically designed notifications on the doors of the toilet cubicles (collectively sampling rooms) the women visited when they gave their routine pregnancy follow-up urine samples. Notifications were also put on the notice boards at the clinics. Furthermore, a separate research disclosure material was available near the sample collection box. Additional information, if required, was made available through dedicated mobile phone and email. Accurate participation rates were not calculable in the units with self-recruitment, because the number of visitors that were actually reached with the study information was unavailable. Therefore, the rates were estimated using the monthly number of patients.

Each sampling room at all research sites had the research disclosure together with instructions for sample treatment available. The sampling rooms were equipped with anonymous informed consent forms, a pen, plastic cups, 5 ml syringes with caps, and fully opaque self-adhesive plastic envelopes. The sample collection box was located outside the rooms for centralized collection. The box had a locked latch that allowed an easy insertion of samples but prevented unauthorized removal. The participants autonomously filled the syringes, put them together with the filled consent form in the plastic envelope, and dropped the envelope to the collection box. The samples were collected two to three times a week.

We recorded the part of week when the samples were collected in two categories: early week = Monday to mid Wednesday, late week = mid Wednesday to Friday. The pregnancy trimester at the time of sample collection was marked by participants in the anonymous consent form in three categories: 1^st^ trimester (gestational weeks 4–12), 2^nd^ trimester (gestational weeks 13–27) and 3^rd^ trimester (gestational weeks 28-).

All samples were tested for ethyl glucuronide using commercially available screening test strips (Confirm Bioscience HETG-105c). The lowest cut-off in these test strips is 300 ng/m. Screening tests results were categorized as positive (no visible line in the test strip result area), uncertain (very light or smudged unclear line in the test strip result area), or negative (visible red line in the test strip result area). Majority of clear negative samples in the screening were discarded after testing according to the research protocol. All positive samples, samples with uncertain screening result and randomly selected 10% of screening negative samples were frozen in -25C and sent to centralized laboratory for quantitative analysis.

Use of tobacco and cannabis products was assessed using urine test strip rapid tests. All HAL clinic samples, test strip positive samples, uncertain samples, and the randomly selected EtG-negative samples were tested for nicotine and cannabis metabolites. For tobacco use the presence of cotinine, a nicotine metabolite, was tested using Confirm Bioscience HDCT-114 tests with a cut-off of 200 ng/mL. For cannabis, the presence of tetrahydrocannabinol (THC) was tested with a commercially available assay from Confirm Biosciences HDTH-114 with a cut-off of 50 ng/mL. Because of temporary unavailability of cotinine and cannabis tests, three EtG-negative HAL samples could not be tested.

As a lower cut-off of 100 ng/mL has been used previously for indicating alcohol exposure in pregnancy [20, 21], those samples that in quantitative laboratory analysis had > 100 ng/mL but < 300 ng/mL were considered separately in further statistical analysis in addition to samples exceeding the EtG content of 300 ng/mL.

### Laboratory analyses

Quantitative EtG measurements were carried out in an SFS-EN ISO 15189:2013–accredited centralized laboratory at the Medical Research Unit, Seinäjoki Central Hospital, Finland using Microgenics DRI EtG immunoassay reagents on Indiko Plus clinical chemistry analyzer (Thermo Fisher Scientific). The measurements were conducted blind to the knowledge of the clinical information and case/control status.

### Statistical analysis

Statistical analysis was done using R 4.1.3 [26]. Figures were produced using Matplotlib [27]. We analyzed the accuracy of the strip tests by calculating the point estimates with 95% confidence intervals for false negative and false positive test results. For the prevalence of positive EtG samples we classified the samples in four groups: HAL antenatal clinic for women with problematic substance use, Lahti central hospital antenatal Clinic (LHC), pooled results from the three user self-recruitment units (USR), and one group encompassing all except the HAL clinic (LCH + USR). We calculated point estimates and 95% confidence intervals for each group. The expected value of positive 300 ng/mL samples in each group was small; thus, we compared the differences in the proportions of positive test samples using two-tailed Fisher’s exact test for count data. We used an odds ratio to quantify the risk ratio between the groups.

Because of the relatively high number of positive 100 ng/mL samples, we did a post-hoc analysis by repeating the groupwise analyses using the laboratory confirmed results with a 100 ng/mL cut-off. To confirm the direction of change between the trimesters, we used one-tailed Fisher’s exact test for count data.

## Results

In this series consisting of 505 urine samples from pregnant women, most samples (443, 87.7%) were clear negatives in EtG screening. All clear positive (*N* = 5), all uncertain (*N* = 57) and 49 assumedly negative samples were frozen in -25C and sent for quantitative confirmatory analysis. There were no uncertain cotinine or cannabis test results.

Participation rate data was available for the two antenatal hospital clinics: 97% in LCH, and 65% in HAL clinic. We do not know how many women were reached with the information and the accurate number of visitors in the USR units, but it was estimated, that the participation rates in these clinics were low (less than 5% of the number of visitors in these units).

Comparisons of the rapid screening test data and quantitative EtG analyses showed no false positives or false negatives in the screening results when the uncertain results were excluded (Table 1). All samples categorized as screening positive had EtG levels above the 300 ng/mL cut-off. All samples categorized as screening negative contained less than 300 ng/mL of EtG. The proportion of uncertain screening results was 11.3% (57/505). Of those 6 (10.5%) contained > 300 ng/mL of EtG and two > 500 ng/mL.

**Table 1:** Comparisons of the data using rapid test strips or quantitative analysis of urine ethylglucuronide

Test strip screening result	Number of samples in comparison	Laboratory result < 100 ng/mL	Laboratory result 100-300 ng/mL	Laboratory result > 300 ng/mL	Laboratory result > 500 ng/mL (included in > 300 ng/mL)
Positive	5	0	0	5	5
Uncertain	57	25	26	6	2
Negative	49	43	6	0	0
Total number	111	68	32	11	7

32/57 (56.1%, CI 43.3%-69.0%) of the samples categorized as uncertain and 6/49 (12.2%, CI 3.8–21.4%) of the samples categorized as screening negative were positive in confirmatory analysis when 100 ng/mL was used as a cut-off.

The prevalence of positive samples (exceeding the level of 300 ng/mL) in the HAL group was 7.3% (CI 1.1%-13.6%). The pooled prevalence in all other groups was 1.4% (CI 0.2%-2.5%). Among those non-selected groups, the LCH group showed higher percentages for positive test results compared to USR (OR 2.1), but the difference was not statistically significant. The HAL group showed higher odds for elevated values than LCH (OR 4.1, *p* = 0.041), USR (OR 8.8, *p* = 0.008), or pooled prevalence of other than HAL groups (OR 5.7, *p* = 0.009). Test data for each group is shown in Table 2. For those samples in which an EtG cut-off of 300 ng/mL was exceeded, LCH saw EtG levels range from 327 ng/mL to 1540 ng/mL, USR from 329 ng/mL to over 500000 ng/mL, and the HAL clinic from 384 ng/mL to 8399 ng/mL.

**Table 2:** Ethylclucuronide (EtG) quantitative findings, smoking status and cannabis use in the study population

Clinic	Samples	EtG Negative	EtG 100-300 ng/mL	EtG > 300 ng/mL	cotinine + /EtG negative	cotinine + / EtG 100-300 ng/mL	cotinine + / EtG > 300 ng/mL	cannabis + /EtG negative	cannabis + / EtG > 100 ng/mL
HAL, antenatal clinic for women with problematic substance use	68	82.4% (56/68)	10.3% (7/68)	7.4% (5/68)	22.6% (12/53)	71.4% (5/7)	100% (5/5)	3.8% (2/53)	17% (2/12)
LCH, Lahti central hospital antenatal clinic	212	92.5% (196/212)	5.7% (12/212)	1.9% (4/212)	25.9% (7/27)	25% (3/12)	50% (2/4)	7.4% (2/27)	0% (0/16)
USR, Helsinki, a community maternity clinic	58	93.1% (54/58)	6.9% (4/58)	0% (0/58)	5.6% (1/18)	0% (0/4)	0% (0/0)	0% (0/18)	0% (0/4)
USR, Lahti, a community maternity clinic	50	92% (46/50)	6% (3/50)	2% (1/50)	10% (1/10)	0% (0/3)	100% (1/1)	0% (0/10)	0% (0/4)
USR, Helsinki Prenatal ultrasound screening unit	117	94% (110/117)	5.1% (6/117)	0.8% (1/117)	0% (0/31)	0% (0/6)	0% (0/1)	0% (0/31)	0% (0/7)
All	505	91.5% (462/505)	6.3% (32/505)	2.2% (11/505)	15.1% (21/139)	25% (8/32)	72.7% (8/11)	2.9% (4/139)	6.3% (2/32)
All except HAL	437	92.9% (406/437)	5.7% (25/437)	1.4% (6/437)	10.5% (9/86)	12% (3/25)	50% (3/6)	2.3% (2/86)	0% (0/25)

*HAL* special antenatal hospital clinic for women with problematic substance use, *LCH* Lahti central hospital antenatal clinic, which is a regular antenatal hospital clinic, *USR* user recruiting units

The prevalence of positive samples exceeding 100 ng/mL (Table 2) in the HAL group was 17.6% (CI 8.6%-26.7%). The pooled prevalence in other groups was 7.1% (CI 4.7%-9.5%). The difference between LCH and USR was not significant (OR 1.1, *p* = 0.8). Though the differences were smaller than those in comparisons with the higher cut-off, the HAL group had again higher risk than LCH (OR 2.6, *p* = 0.021), USR (OR 3.0, *p* = 0.014), or pooled prevalence of other than HAL groups (OR 2.8, *p* = 0.008).

**Fig. 1:**
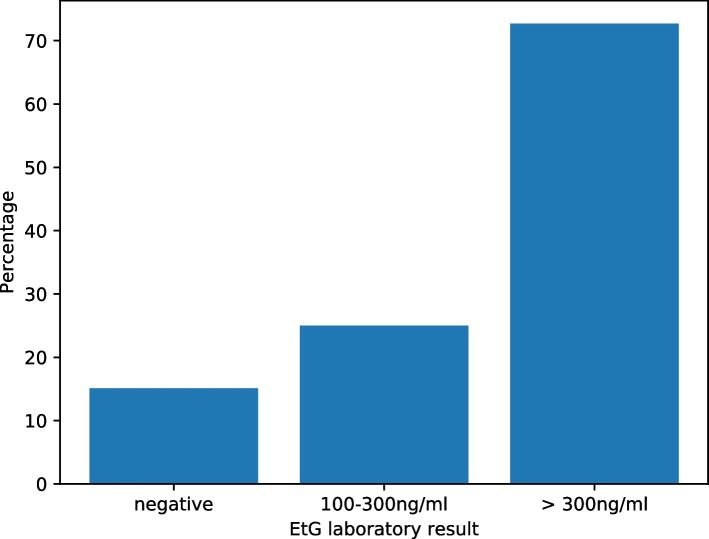
Smoking status in mothers with different levels of ethylglucuronide (EtG) in urine

The prevalence of positive cotinine samples was 58.5% (CI 46.5%-70.4%) in the HAL group and 12.8% (CI 6.8%-18.9%) in pooled other groups. Cotinine test was positive more often in samples exceeding EtG levels of 300 ng/mL (OR 7.5, *p* = 0.002). Using a 100 ng/mL cut-off, the difference was not significant (Table 2 and Fig. 1). In this material, there were only six cannabis positive samples of those tested, four of them were from HAL and other two from LCH, meaning that no cannabis users participated in the study in the self-recruiting units. 66.7% (4/6) of cannabis users had negative EtG and 33.3% (2/6) had EtG levels above 100 ng/mL (Table 2).

45.3% (229/505) of the samples were given in the 3^rd^ trimester, which is the time when most prenatal visits take place. The fetal ultrasound screening unit differed from other units because the screenings are scheduled in gestational weeks 11 + 0 -13 + 6 and in weeks 19 -21. Only 15.0% (76/505) of the samples were given in the 1^st^ trimester. In all groups the proportion of positive samples decreased from the 1^st^ to the 2^nd^ trimester. In the LCH and USR groups the proportion again increased from the 1^st^ to the 3^rd^ trimester, though the differences were not statistically significant. However, post-hoc analysis using a 100 ng/mL cut-off revealed that the proportion increased in the 3^rd^ trimester in LCH (OR 6.4, *p* = 0.033) and the pooled group of other than HAL samples (OR 3.8, *p* = 0.004). Trimester had no significant effect on the proportions of positive cotinine or cannabis tests.

59.2% (299/505) of samples were given in early part of the week. Only in the HAL samples were there differences in the percentages of positive samples between early 23.3% (7/30) and late 13.2% (5/38) week, but the differences did not reach statistical significance.

## Discussion

There is a clear need for objective biomarkers to identify alcohol drinking during pregnancy. The present work employing rapid screening techniques and quantitative determinations of ethyl glucuronide (EtG) from urine samples of pregnant mothers indicates that the use of easy and inexpensive biomarker screening techniques may prove to be of value in recognizing and following those at risk for alcohol-induced harm for fetal development. The occurrence of EtG positive samples in our study also supports the usefulness of biomarker-based screening in providing additional value in detecting alcohol consumption during pregnancy. Currently self-reporting and AUDIT (Alcohol Use Disorder Identification Test) is used for screening of alcohol use during pregnancy. All women who either had high AUDIT score before pregnancy or continue alcohol use during pregnancy should be referred to HAL clinics according to recommendations [4]. However, compared to the number of women who according to anonymous surveys continue using alcohol during pregnancy [7-9] or the number of EtG positive samples in this study, only a small fraction of the pregnant population with alcohol consumption during pregnancy is identified and attend HAL clinics.

The lowest available EtG cut-off in commercially available urine EtG rapid screening tests (300 ng/mL) appears to be too high for screening during pregnancy. The commercial tests are aimed at screening harmful alcohol drinking among non-pregnant population and used for forensic purposes. Accordingly, only higher clinically relevant EtG concentration levels are of interest. Using lower cut-off increases the theoretical risk of detecting ethanol exposure from other sources beside conscious consumption of alcohol. However, in the case of fetal alcohol exposure, it is not relevant how the alcohol has entered the mother’s bloodstream, if there is significant amount of it. Since there is no known safe level for fetal alcohol exposure during pregnancy, a cut-off of 100 ng/mL has been used in an earlier study showing detectable morphological changes by the 2^nd^ trimester ultrasound in those exceeding that limit [21]. For those reasons, we chose to also include in confirmatory quantitative analysis all samples categorized as uncertain in screening and, also randomly selected screening negative samples to test the validity of the current screening test. There were six samples categorized as uncertain with EtG exceeding 300 ng/mL. The lower cut-off of 100 ng/mL was exceeded in 32/57 (56.1%) of samples categorized as uncertain and in 6/49 (12.2%) of quantitatively analyzed screening negative samples.

One urine test is able to reveal alcohol use only during the preceding few days prior to sampling, and amount and frequency of alcohol use during pregnancy may vary. For that reason, repeated testing during pregnancy would be needed for effective screening purposes. A positive test result should warrant in-depth counseling and follow-up including repeated testing. When used in primary care maternity clinics, a positive test result could be one indication for referral to special antenatal clinics for pregnant women with substance abuse problems when deemed necessary after counseling.

In clinics treating pregnant women with known substance abuse problems, rapid EtG urine screening tests would provide an easy additional method for follow-up of treatment effectiveness and adherence to treatment. Currently, urine testing for screening illegal drugs and ethanol breath analysis are commonly used clinical tools. The short half-life of ethanol, however, hampers the use of ethanol analyses for detecting recent ethanol intake. In addition to EtG, a blood test for phosphatidylethanol (PEth), another specific metabolite of ethanol, can be used to detect alcohol use from the previous days or even weeks prior to sampling [28] but is more invasive and expensive than rapid urine EtG screening.

All screening requires informed consent with a mutual understanding with the pregnant woman. In Finland nearly all pregnant women use primary care maternity clinics and a majority participate in voluntary screening for fetal chromosomal and structural abnormalities, metabolic disorders, and maternal infections. Adding urine EtG screening to routine programs would be expected to benefit both the mother and the child. In a Swedish study yearly societal cost was estimated as 76 000€ per one child and 110 000€ per one adult with FAS [29]. Thus, by a direct calculation the screening costing approximately 1 million euros yearly would be cost-effective if it helped to save only 9–13 persons yearly from FAS.

Yearly, 700–1000 (1.4%–2% of all) pregnant women in Finland attend HAL clinics [30], although all who continue alcohol use during pregnancy should be referred according to guidelines [4] and more women than before are currently referred to HAL clinics for drug use and social problems compared to alcohol use [30]. As expected, HAL clinic samples were more often positive for EtG compared to other sample collection units. Also, more HAL samples were positive for EtG on Monday to mid-Wednesday compared to samples obtained between mid-Wednesday to Friday suggesting that more alcohol consumption occurs during weekends consisting probably of binge-type drinking. Tailored interventions for pregnant women with substance use disorders have been previously shown to be beneficial and cost-effective [31]. Because in HAL clinic samples the fractions of both EtG > 300 ng/mL positive samples and cotinine positive samples were lower in the 3rd trimester than in the 1st trimester, our data supports the impact of outpatient interventions at HAL clinics.

It is noteworthy that, apart from the HAL group, the proportion of positive EtG samples increased statistically significantly in the 3^rd^ trimester. This could signal that there remains a misconception about the effects of alcohol during different stages of the pregnancy or a change in the life situation. Because the central nervous system develops through the pregnancy, there is no safe timing for alcohol consumption. It remains important to ask about alcohol consumption at every stage of the pregnancy, not only in the beginning.

### Strengths of the study

The strengths of our study include recruitment of the participants from different maternity care and antenatal clinics in a consecutive manner, which should provide a real-life setting. The samples were collected anonymously which may help to reduce bias in the detection of positive samples. Analyses of EtG were carried out with both rapid screening tests and confirmatory laboratory analyses.

### Limitations of the study

In urine EtG analysis there is a theoretical possibility of both false negative [32] and false positive [33] results, especially in cases of urinary tract infections. However, use of alcohol containing mouthwash and heavy use of hand sanitizer are not likely to cause positive results in pregnant women [20]. There are also other possible extraneous sources of alcohol exposure that might in theory cause positive EtG results if those alcohol containing products are inhaled or ingested in substantial quantities. However, for the fetal development the amount and timing of alcohol exposure is likely to be more relevant than the route of exposure. Low participation rate in user self-recruiting units caused probable bias in results, because there were only two screening positive samples, a very low percentage of tobacco users and no cannabis users in those who participated through such recruiting procedures. Still, in quantitative analysis of samples including uncertain screening results and randomly selected screening negative samples and using lower cut-off, 6.7% of samples in those units exceeded 100 ng/mL.

## Conclusions

The rapid urine analyses for EtG provide a new, easy, and cost-effective approach for health care to recognize women who use alcohol during pregnancy. The rate of positive EtG results in this study confirmed that there still are significant numbers of women with alcohol consumption during pregnancy. The rapid urine EtG testing could be used either as part of routine pregnancy health checks or when regular alcohol consumption is suspected but denied by the pregnant woman. It also provides a tool for follow-up of pregnant women with known alcohol consumption. More sensitive urine screening tests with a EtG cut-off of 100 ng/mL are needed for screening alcohol exposure during pregnancy, which has remained as a major public health challenge throughout the world.

## Data Availability

The datasets generated and analyzed during the current study are available from the corresponding author on reasonable request.

## Abbreviations

ARND: Alcohol-related neurodevelopmental disorder
EtG: Ethyl glucuronide
FAS: Fetal alcohol syndrome
FASD: Fetal alcohol spectrum disorder
HAL: A tertiary specialist antenatal clinic for pregnant women with problematic substance use at Women’s Hospital in Helsinki
LCH: Lahti central hospital specialist antenatal clinic
PAE: Prenatal alcohol exposure
PEth: Phosphatidylethanol
THC: Tetrahydrocannabinol
USR: User self-recruiting units

## References

[1] Hoyme HE, Kalberg WO, Elliott AJ, Blankenship J, Buckley D, Marais AS, Manning MA, Robinson LK, Adam MP, Abdul-Rahman O, Jewett T, Coles CD, Chambers C, Jones KL, Adnams CM, Shah PE, Riley EP, Charness ME, Warren KR, May PA (2016). updated clinical guidelines for diagnosing fetal alcohol spectrum disorders. Pediatrics.

[2] Mattson SN, Bernes GA, Doyle LR (2019). Fetal alcohol spectrum disorders: a review of the neurobehavioral deficits associated with prenatal alcohol exposure. Alcohol Clin Exp Res.

[3] World Health Organization. "Guidelines for the identification and management of substance use and substance use disorders in pregnancy." World Health Organization. 2014. https://www.who.int/publications/i/item/9789241548731. Accessed 9 June 2023. 24783312

[4] Klemetti R, Hakulinen-Viitanen T. Editors (2013). Äitiysneuvolaopas-suosituksia äitiysneuvolatoimintaan. Terveyden ja hyvinvoinnin laitos. Juvenes Print – Suomen Yliopistopaino Oy. https://urn.fi/URN:ISBN:978-952-245-972-5. Accessed 9 June 2023.

[5] Lange  S, Shield  K, Koren  G, Rehm  J, Popova  S (2014). A comparison of the prevalence of prenatal alcohol exposure obtained via maternal self-reports versus meconium testing: a systematic literature review and meta-analysis. BMC Pregnancy Childbirth.

[6] Finnish Institute of Health and Welfare. Yearbook of alcohol and drug statistics 2021. Official Statistics of Finland. Suomen virallinen tilasto (SVT) : SVT_PTVK_2021. https://urn.fi/URN:ISBN:978-952-343-817-0. Accessed 10 Dec 2022.

[7] Mårdby AC, Lupattelli A, Hensing G, Nordeng H (2017). Consumption of alcohol during pregnancy-a multinational European study. Women and Birth : Journal of the Australian College of Midwives.

[8] Komulainen J, Lepistö S, Helminen M, Kylmä J, Paavilainen E. Tulevien vanhempien alkoholinkäyttö raskausaikana. Sosiaalilääketieteellinen Aikakauslehti. 2017;54(4).

[9] Voutilainen T, Rysä J, Keski-Nisula L, Kärkkäinen O (2022). Self-reported alcohol consumption of pregnant women and their partners correlates both before and during pregnancy: a cohort study with 21,472 singleton pregnancies. Alcohol Clin Exp Res.

[10] Eichler A, Grunitz J, Grimm J (2016). Did you drink alcohol during pregnancy? Inaccuracy and discontinuity of women's self-reports: On the way to establish meconium ethyl glucuronide (EtG) as a biomarker for alcohol consumption during pregnancy. Alcohol (Fayetteville, N.Y.).

[11] Hemingway SJA, Bledsoe JM, Brooks A (2019). Comparison of the 4-Digit Code, Canadian 2015, Australian 2016 and Hoyme 2016 fetal alcohol spectrum disorder diagnostic guidelines. Advances in Pediatric Research.

[12] May PA, Chambers CD, Kalberg WO (2018). Prevalence of fetal alcohol spectrum disorders in 4 US communities. JAMA.

[13] Petković G, Barišić I (2013). Prevalence of fetal alcohol syndrome and maternal characteristics in a sample of schoolchildren from a rural province of Croatia. Int J Environ Res Public Health.

[14] Okulicz-Kozaryn K, Borkowska M, Brzózka K (2017). FASD prevalence among schoolchildren in Poland. Journal of Applied Research in Intellectual Disabilities : JARID.

[15] Bakhireva LN, Savage DD. Focus on: biomarkers of fetal alcohol exposure and fetal alcohol effects. Alcohol Res Health. 2011;34(1):56–63. PMC3860558 23580042

[16] Niemelä O, Niemelä S, Ritvanen A (2016). Assays of gamma-glutamyl transferase and carbohydrate-deficient transferrin combination from maternal serum improve the detection of prenatal alcohol exposure. Alcohol Clin Exp Res.

[17] Helander A, Böttcher M, Fehr C, Dahmen N, Beck O (2009). Detection times for urinary ethyl glucuronide and ethyl sulfate in heavy drinkers during alcohol detoxification. Alcohol and Alcoholism (Oxford, Oxfordshire).

[18] Høiseth G, Bernard JP, Stephanson N (2008). Comparison between the urinary alcohol markers EtG, EtS, and GTOL/5-HIAA in a controlled drinking experiment. Alcohol and Alcoholism (Oxford, Oxfordshire).

[19] McDonell MG, Skalisky J, Leickly E (2015). Using ethyl glucuronide in urine to detect light and heavy drinking in alcohol dependent outpatients. Drug Alcohol Depend.

[20] Ondersma SJ, Beatty JR, Rosano TG (2016). Commercial Ethyl Glucuronide (EtG) and Ethyl Sulfate (EtS) testing is not vulnerable to incidental alcohol exposure in pregnant women. Subst Use Misuse.

[21] Ferraguti G, Merlino L, Battagliese G (2020). Fetus morphology changes by second-trimester ultrasound in pregnant women drinking alcohol. Addiction Biology.

[22] Hamułka J, Zielińska MA, Chądzyńska K (2018). The combined effects of alcohol and tobacco use during pregnancy on birth outcomes. Rocz Panstw Zakl Hig.

[23] Lange S, Probst C, Quere M, Rehm J, Popova S (2015). Alcohol use, smoking and their co-occurrence during pregnancy among Canadian women, 2003 to 2011/12. Addict Behav.

[24] Alvik A, Heyerdahl S, Haldorsen T, Lindemann R (2006). Alcohol use before and during pregnancy: a population-based study. Acta Obstet Gynecol Scand.

[25] Sotkanet. Sotkanet.fi Statistics and Indicator Bank. The Finnish Institute for Health and Welfare 2005–2022. Statistical information on welfare and health in Finland. Online Statistical Service. 2022. https://sotkanet.fi/sotkanet/en/index. Accessed 10 Oct 2022.

[26] R Core Team (2022). R: A Language and Environment for Statistical Computing. R Foundation for Statistical Computing, Vienna, Austria. https://www.R-project.org/

[27] Hunter JD (2007). Matplotlib: A 2D graphics environment. Computing in Science & Engineering.

[28] Neumann J, Beck O, Böttcher M (2021). Phosphatidylethanol, ethyl glucuronide and ethanol in blood as complementary biomarkers for alcohol consumption. Journal of Mass Spectrometry and Advances in the Clinical lab.

[29] Ericson L, Magnusson L, Hovstadius B (2017). Societal costs of fetal alcohol syndrome in Sweden. The European Journal of Health Economics : HEPAC : Health Economics in Prevention and Care.

[30] Arponen A. Päihteitä käyttävien raskaana olevien naisten ja vauvaperheiden palvelut: Nykytila ja muutokset viiden vuoden (2016–2020) seurantajaksolla. 2021. Julkari. https://urn.fi/URN:ISBN:978-952-343-783-8. Accessed 9 June 2023.

[31] Metz V, Köchl B, Fischer G (2012). Should pregnant women with substance use disorders be managed differently?. Neuropsychiatry.

[32] Helander A, Dahl H (2005). Urinary tract infection: a risk factor for false-negative urinary ethyl glucuronide but not ethyl sulfate in the detection of recent alcohol consumption. Clin Chem.

[33] Helander A, Olsson I, Dahl H (2007). Postcollection synthesis of ethyl glucuronide by bacteria in urine may cause false identification of alcohol consumption. Clin Chem.

